# Updates in the management of cranial dural arteriovenous fistula

**DOI:** 10.1136/svn-2019-000269

**Published:** 2019-11-21

**Authors:** Humain Baharvahdat, Yinn Cher Ooi, Wi Jin Kim, Ashkan Mowla, Alexander L Coon, Geoffrey P Colby

**Affiliations:** 1 Neurosurgery, Ronald Reagan UCLA Medical Center, Los Angeles, California, USA; 2 Neurointerventional Radiology, UCLA, Los Angeles, California, USA; 3 Western Neuro, Carondelet Health Network, Tucson, Arizona, USA

**Keywords:** dural arteriovenous fistulas, dAVF, embolisation, transarterial, transvenous

## Abstract

Dural arteriovenous fistula (dAVF) accounts for approximately 10% of all intracranial vascular malformations. While they can be benign lesions, the presence of retrograde venous drainage and cortical venous reflux makes the natural course of these lesions aggressive high risk of haemorrhage, neurological injury and mortality. Endovascular treatment is often the first line of treatment for dAVF. Both transarterial and transvenous approaches are used to cure dAVF. The selection of treatment approach depends on the angioarchitecture of the dAVF, the location, the direction of venous flow. Surgery and, to a lesser extent, stereotactic radiosurgery are used when endovascular approaches are impossible or unsuccessful.

## Introduction

Dural arteriovenous fistulas (dAVF), also referred to as dural arteriovenous malformations (AVM), are abnormal shunts between the arterial and venous systems located within the dura. The aetiology of the dAVF is unclear in many instances; however, they are thought to be acquired after trauma, surgery, venous stenosis or sinus thrombosis. Cranial dAVFs most commonly occur by dural venous sinuses.^1 2^

Several classification systems have been developed to categorise dAVFs, specifically by the pattern of venous flow.^3 4^ Borden and colleagues organised dAVFs into three groups (table 1): type I dAVFs drain directly into venous sinus; type II dAVFs drain into venous sinuses but also have retrograde drainage into subarachnoid (cortical) veins; and type III dAVFs drain directly into subarachnoid veins.^4^ Conversely, Cognard and colleagues organised dAVF into five groups (table 1): type I dAVFs drain directly into venous sinus with anterograde flow; type IIa dAVFs drain into the main sinus (anterograde) with additional retrograde reflux into an associated venous sinus; type IIb dAVFs drain into venous sinus in anterograde flow with reflux into a cortical vein; type IIa+b dAVFs drain into the main sinus with reflux into both associated venous sinus and cortical vein in retrograde flow; type III and type IV dAVFs drain directly into cortical veins either without (type III) or with (type IV) venous ectasia; type V dAVFs drain into the spinal perimedullary venous system. In general, dAVFs without cortical venous reflux (CVR) (Cognard type I or IIa or Borden type I) are considered benign^3^ and are thought to have only a 2% risk of developing CVR.^5^ dAVFs with persistent CVR (Cognard type IIb-V or Borden types II and III) are aggressive and have been found to have an annual mortality of 10.4% with an annual haemorrhagic risk of 8.1% and an annual non-haemorrhagic neurological deficit risk of 6.9%.^6^ Following an initial haemorrhage, rebleeding can be as high as 35% in the first 2 weeks.^7^

**Table 1:** Classification of dAVFs: Borden versus Cognard

Natural course	Borden classification			Cognard classification			
	Type	Venous drainage site	CVR	Type	Venous drainage site	Flow pattern in sinus	CVR
Benign	I	Dural sinus	No	I	Dural sinus	Antegrade	No
Benign				IIa	Dural sinus	Retrograde	No
Aggressive	II	Dural sinus	Yes	IIb	Dural sinus	Antegrade	Yes
Aggressive				IIa+b	Dural sinus	Retrograde	Yes
Aggressive	III	Cortical vein	Yes	III	Cortical vein		Yes without venous ectasia
Aggressive				IV	Cortical vein		Yes with venous ectasia
Aggressive				V	Cortical vein with spinal medullary drainage		Yes

CVR, cortical venous reflux; dAVF, dural arteriovenous fistula.

The location of the fistula and its subsequent disruption of normal venous drainage can cause changes in flow dynamics to produce symptoms. Presenting symptoms are variable depending on the location of the fistula and can include pulsatile tinnitus, bruit, headaches, visual changes, alterations in mental status, seizure, myelopathy, cranial nerve palsies and motor or sensory deficits. Approximately 20%–33% of dAVFs present with intracranial haemorrhage.^2 6^

For diagnosis, CT and MRI are usually unremarkable in dAVF. CT scan is often useful for determining haemorrhage or oedema if present. MRI may also show indirect signs of venous hypertension or CVR, such as pial vein engorgement, dilated venous pouch or abnormal vascular enhancement.^8^ Susceptibility-weighted imaging can clarify arteriovenous shunting of dAVF by demonstrating hyperintense venous signal due to rapid wash-in of oxygenated blood.^8^ Dedicated vascular imaging is considered standard for diagnosis of a fistula. Brain CT angiography (CTA)/CT venography or MR angiography (MRA)/MR venography can demonstrate asymmetric sinus, engorged arterial or venous vessels and enhanced transosseous vessels. It can be helpful for treatment planning by defining the relation between the dAVF shunt and skull anatomy.^8^ Negative brain CTA or MRA does not completely exclude the diagnosis of dAVF. Cerebral digital subtraction angiography (DSA) is the gold standard imaging modality to detect and best characterise dAVF.^8^ A full DSA, including internal carotid arteries (ICA), external carotid arteries (ECA) and both vertebral arteries, is usually required to assess dAVF. Superselective evaluation of smaller arteries is also helpful to clarify particular arterial contributions and the location of the fistula/shunt.

Because of the benign nature, dAVFs without CVR (grades I and II) are often managed conservatively. Treatment can be palliative for patients who suffer from incapacitant tinnitus, ocular symptoms or severe headache. Compression therapy is sometimes used as adjunct treatment for dAVF without CVR. Compression of ipsilateral carotid artery or occipital artery is performed by contralateral hand three times a day. Depending on the location and extent of the dAVF, this management was reported to cure dAVFs in 20%–30% of cases.^9 10^ Various treatment modalities are used to manage aggressive dAVFs, including endovascular techniques, surgery, radiosurgery or a combination of these treatments.

## Endovascular approach

**Figure 1:**
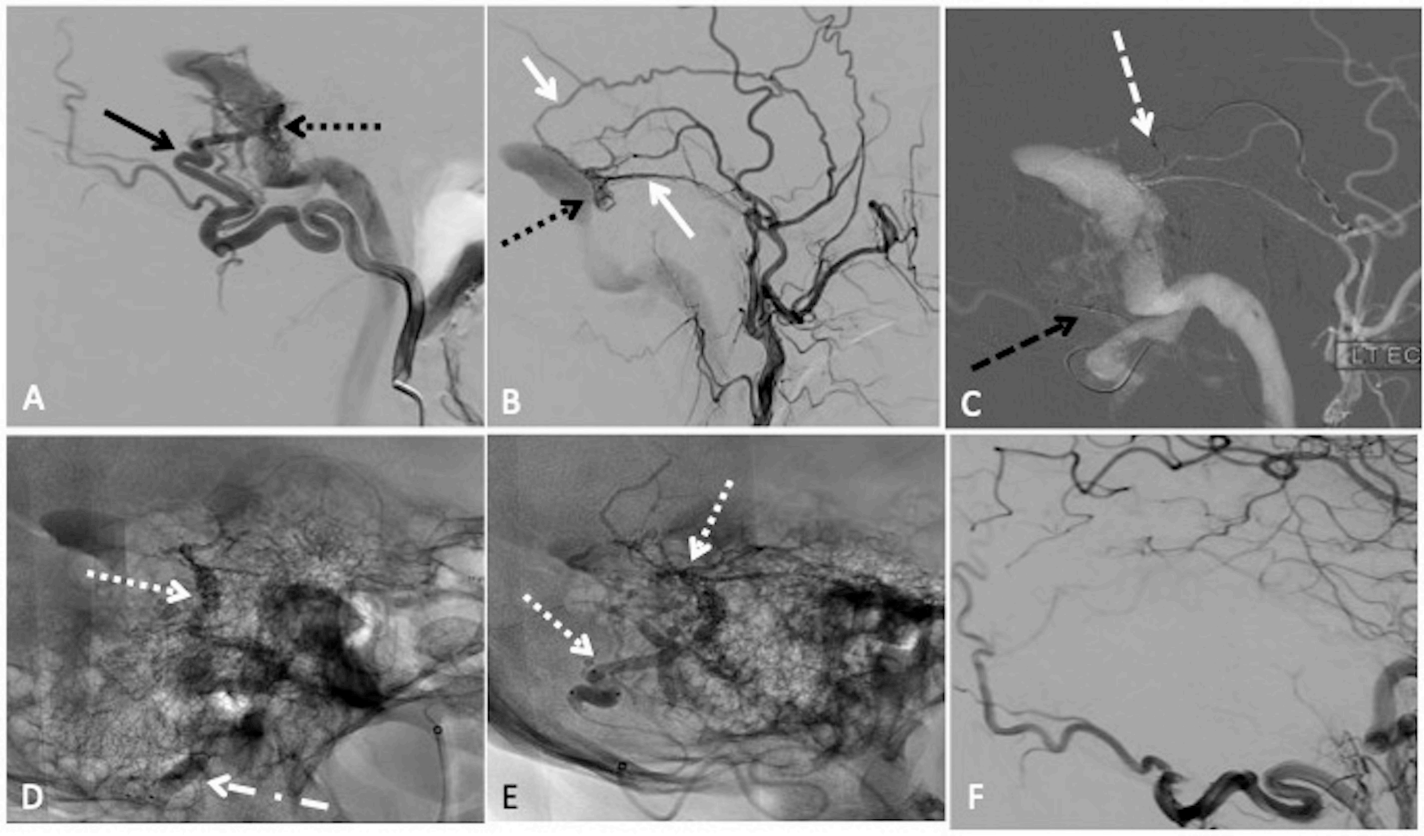
Transarterial embolisation with two-microcatheter technique of a left sigmoid sinus dural arteriovenous fistula (dAVF) with precipitating hydrophobic injectable liquid (PHIL). (A) Lateral projection pre-embolisation angiogram, selective left occipital artery (OA) injection and (B) left main trunk external carotid artery injection showing multiple arterial feeders arising from the OA (solid black arrow) and middle meningeal artery (MMA) (solid white arrow), draining into a common channel (dotted black arrow). (C) Lateral road map angiogram showing dual microcatheter technique with positioning of Headway Duo microcatheter within the MMA (dashed white arrow) and Scepter C 4×10 mm balloon microcatheter in the OA (dashed black arrow). (D) Unsubtracted lateral view showing PHIL cast (dotted white arrow) after single infusion through the MMA with inflation of the Scepter balloon (dotted dashed black arrow) to reduce dAVF flow. (E) Final PHIL cast (dotted white arrow) following infusion through the MMA and OA. (F) Final lateral digital subtraction angiography (DSA), left common carotid injection showing no residual dAVF.

**Figure 2:**
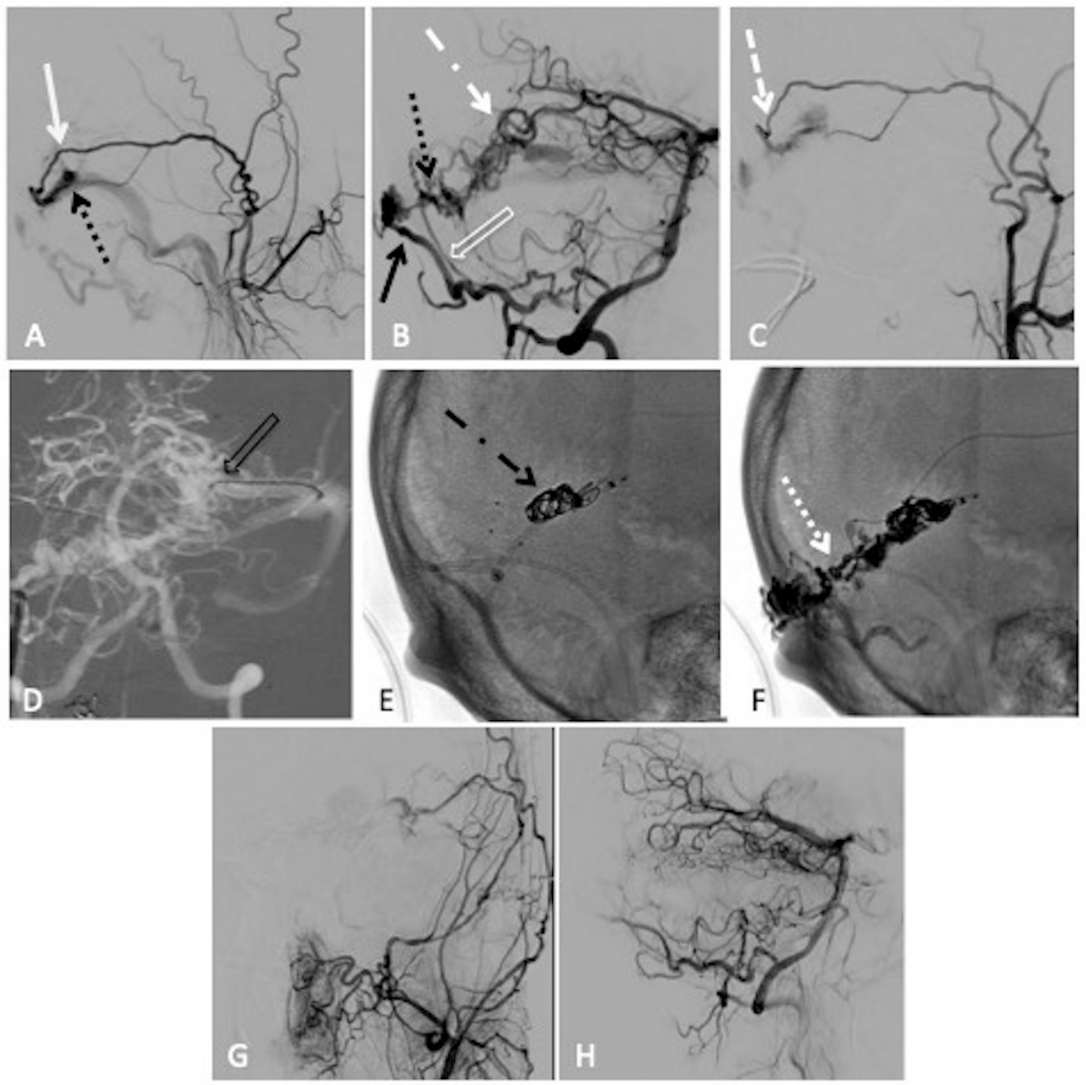
Combined transarterial and transvenous embolisation of a left transverse sinus dural arteriovenous fistula (dAVF) with Onyx and microcoils. (A) Lateral projection pre-embolisation angiogram, selective left external carotid artery (ECA) injection and (B) right vertebral artery (VA) injection showing multiple arterial feeders arising from the left middle meningeal artery (MMA) (solid white arrow), right occipital artery (solid black arrow), right posterior meningeal artery (clear white arrow) and left artery of Davidoff and Schechter (dotted white dashed arrow), draining into a common channel (dotted black arrow). (C) Left ECA injection showing Scepter XC 4×11 mm microcatheter positioned within the MMA (dashed white arrow). (D) Anteroposterior (AP) road map showing Echelon 14 microcatheter positioned through the left transverse sinus and within the venous pouch (clear black arrow). Unsubtracted lateral view, (E) showing successful deployment of microcoils within the venous pouch (dotted black dashed arrow), (F) showing the Onyx cast after successful infusion through the MMA (dotted white arrow). Postembolisation lateral angiogram, (G) right VA injection and (H) left common carotid artery injection showing no residual dAVF.

Endovascular approach is the first-line treatment for most dAVFs. The mainstay for endovascular treatment involves embolisation of the fistulous connection and its venous components while preventing adverse effects.^11^ Inappropriate embolisation of the fistulous connection and venous portions could cause sudden changes in the flow dynamics and potentially worsen cortical venous flow. Therefore, it is imperative to have an in-depth understanding of the fistula and its arterial and venous components prior to initiating treatment. The fistulous connection can be approached by either transarterial or transvenous methods. Although in the past, detachable balloons, polyvinyl alcohol, silk sutures and microspheres were used for treatment of cerebral AVM and dAVFs they have been widely replaced by current embolic agents, including *n*-butyl-2-cyanoacrylate (*n*-BCA, glue, Trufill, DePuy Synthes, Raynham, MA), Onyx (ev3 Endovascular, Irvine, CA), Squid (Emboflu, Switzerland), precipitating hydrophobic injectable liquid (PHIL; MicroVention, Aliso Viejo, California) and detachable microcoils. Recent advancements have introduced newer embolic agents, such as PHIL^12^ and Squid (Emboflu),^13^ and flow diverters, such as the pipeline embolisation device (Medtronic Neurovascular, Irvine, California), to treat AVFs and inhibit fistula recanalisation in special scenarios,^14^ as described by Castãno *et al* with the treatment of two Barrow type B indirect carotid cavernous fistula (CCF), a version of dAVF. Classic approaches described for endovascular treatment of dAVFs include transarterial, transvenous or a combination of both techniques. Figures 1 and 2 are two illustrative cases that highlight the transarterial and combination of transarterial and transvenous approach, respectively.

## Transarterial embolisation

A transarterial approach is the preferred treatment for high-grade dAVFs with direct cortical venous drainage or in cases in which transvenous approach is limited. Advantages of transarterial embolisation include decreased chance of flow redirection into an alternate venous pathway, ability to save functional venous system, avoidance of post-treatment de novo dAVF formation from venous hypertension and decreased complications specific to commonly used transvenous approaches (eg, abducens nerve palsy from catheterisation of the superior petrosal sinus, and so on).^15^ Transarterial approaches are often done under general anaesthesia with motor paralysis to decrease patient motion. During the procedure, patients are anticoagulated with heparin. Heparinised/non-heparinised saline flushes are additionally used to prevent catheter-associated thrombosis and embolic events. Various imaging techniques, including superselective microcatheter angiography, three-dimensional rotational angiography and high-resolution flat-panel CT, are often used to obtain high-resolution images throughout the procedure.^8^ In this approach, different liquid embolic agents are used: Cyanoacrylic glue, Onyx, Squid and PHIL.

In transarterial approaches, microcatheters are tracked over microwires to distal locations in feeding arteries, with the goal of getting the microcatheter as close to the fistula connection as possible. Injection of an embolic agent through a compatible dual-lumen balloon catheter can be used in certain scenarios to help prevent proximal reflux of the embolic agent. 

### Cyanoacrylic glue

Cyanoacrylate adhesives are widely used for the embolisation of dAVFs with high flow.^15–17^ The most common agent is *n*-BCA (commonly referred to as ‘glue’), a liquid agent that quickly solidifies when it comes in contact with ionic substances, including blood. Ethiodol (ethiodised oil), tantalum or tungsten powder is often added to *n*-BCA to make the mixture radiopaque and identifiable on fluoroscopy.^16^ The concentration of *n*-BCA is an important consideration as it changes the extent of migration or penetration of the product before polymerisation. A mixture with high *n*-BCA-to-Ethiodol ratio (high concentration of glue) polymerises more rapidly and therefore embolises more proximal targets. A mixture with low *n*-BCA-to-Ethiodol ratio travels further and can achieve more distal penetration. Typically, concentrations of 25%–33% *n*-BCA are used.

Prior to embolisation, the microcatheter must be placed as close to the fistula target as possible. Ideally, the microcatheter should be wedged into the feeding artery to create a flow arrest, which helps successfully deliver the glue to the fistulous site and allows for permeation of the glue to the fistulous collateral networks. Nelson and colleagues have reported on this technique and demonstrated complete occlusion of the fistula in 23 dAVF embolisations.^15^ Before embolising with glue, microcatheter angiography runs are obtained from the final microcatheter position to help establish the optimal glue concentration needed for the embolisation. Prior to injecting the *n*-BCA and Ethiodol mixture, the microcatheter is primed by flushing with non-ionic solution, such as 5% dextrose, to prevent any glue polymerisation within the catheter delivery system. The glue is then injected under direct DSA or negative road map as either a continuous column or a bolus.^18^ Depending on the position of the microcatheter and the distance to the intended target, 5% dextrose solution can be simultaneously injected through the guide or distal intracranial support catheter to promote more distal migration of the glue.^17^ After embolisation with *n*-BCA, it is important to rapidly remove the microcatheter to prevent the catheter from being glued to the vessel.

### Onyx

Onyx is a liquid mixture of ethylene–vinyl alcohol copolymer suspended in dimethyl sulfoxide (DMSO). All materials involved in the procedure (catheters, syringes, and so on) must be DMSO compatible. Similar to *n*-BCA, tantalum powder is added for radio-opacity. The Onyx mixture must be shaken for 20 min to evenly distribute the tantalum and obtain uniform radio-opacity before use.^19^ Onyx precipitates into a viscous substance with a characteristic ‘lavalike’ flow pattern on contact with blood to cause vessel occlusion.

Onyx can be the preferred treatment option based on the type and location of the cerebral dAVF. Although the best therapeutic approach for Cognard type II dural fistulas, with or without CVR, is transvenous approach, transarterial embolisation with Onyx can be useful to avoid sacrificing the draining venous sinus in certain cases.^20–23^ For higher grade dAVF (Cognard types III–V), transarterial approach with Onyx has demonstrated much success.^24–26^ Compared with *n*-BCA glue, Onyx is less operator dependent, does not require a wedged microcatheter position, as the Onyx itself will form a plug that helps prevent reflux. The ability to perform prolonged injections allows for embolisation of multiple feeders forming complex vessel networks from a single injection, especially when venous access is limited.^11 24–26^ Endovascular treatment with Onyx has shown to achieve a higher rate of dAVF cure than *n*-BCA.^27^ Although transarterial embolisation with Onyx can be used to treat indirect CCF, transvenous approach is preferred to avoid the risk of injury to cranial nerves and penetration into extracranial or intracranial dangerous anastomoses.^20 23^

After establishing an in-depth understanding of the anatomy and flow dynamics of the fistula, the microcatheter must be primed/flushed with DMSO, prior to injecting Onyx. The Onyx can then be injected under direct visualisation, using a double road map fluoroscopy to identify any premature leakage.^28^ The speed of injection can be customised to optimise vessel penetration, direction and reflux. Once reflux is observed, injection should be stopped for approximately 30–90 s to allow the Onyx to solidify before proceeding with further injection. As reflux creates a desirable plug around the microcatheter it can be used to further push Onyx into the fistula. Creation of the plug is an important part of the technique, particularly if the microcatheter tip position is more proximal.

Several complications can occur with Onyx injection and reflux. Uncontrolled or excessive reflux of Onyx can cause retrograde filling and cause inadvertent obliteration of proximal vasculature and branch points. Additionally, delayed injection after reflux can lead to occlusion or entrapment of the microcatheter. Multiple control angiograms are often obtained throughout the procedure to carefully monitor the progression of the embolisation. Because much precision is required to inject Onyx, it is not uncommon for treatments through a single pedicle to last more than an hour or to separate the embolisation into multiple stages.

After the embolisation the microcatheter can be removed from the Onyx cast with continuous gentle traction. The distal microcatheter tip can be cut and left in place if it is entrapped by the Onyx.^21^ Detachable tip microcatheters, such as Apollo (Medtronic Neurovascular), were designed to limit complications during catheter withdrawal. The Apollo has a detachable catheter tip that is released on reaching a threshold withdrawal force, allowing for longer Onyx injections without concern for catheter entrapment.

### PHIL and Squid

PHIL (MicroVention) is an iodinated copolymer dissolved in DMSO that precipitates to form a non-adhesive material when comes into contact with blood. The iodine component of PHIL provides radio-opacity without the need for tantalum, in which results are thought to result in less artifact on CT and eliminate the need for preprocedural mixing that is needed for Onyx. PHIL can be used in three concentrations, with the lowest concentration being used most often for dAVF embolisations.

PHIL is currently not available for commercial use in the USA, but there is an ongoing active trial to evaluate its effectiveness in treatment of dAVFs. Leyon and colleagues described using PHIL in eight cases to treat five cranial and three spinal dAVFs with either Apollo (Medtronic) or Marathon (Medtronic) microcatheter delivery systems.^29^ Lamin and colleagues are also reported using PHIL in 30 procedures with Apollo, Marathon or Headway Duo (MicroVention) microcatheter delivery systems.^12^

Squid is another liquid embolic agent for treatment of dAVF, but currently not available in the USA. Squid is an ethylene vinyl alcohol copolymer available in two versions: Squid 12 and Squid 18. Squid has 30% less tantalum than Onyx and has micronised tantalum, which may help better visualise structures behind the embolised material and provide a more homogeneous solution than Onyx.^13 30^ Akmangit and colleagues reported a case series of using Squid to treat nine dAVFs with Sonic (Balt, Montmorency, France), another detachable microcatheter delivery system.^13^ Much like the Onyx, the Squid works in a ‘plug and push’ method. In their case series, Akmangit and colleagues described^13^ using Squid 18, which has higher density, for the initial plug formation and using Squid 12, which has lower viscosity, for distal penetration.

### Flow diversion

While flow-diverting stents have been used to treat direct CCFs,^31^ there is limited efficacy for treatment of the most common dAVF because of lesion complexity including multiple arterial feeders’ origins (ECAs, ICAs and vertebral arteries). However, Castãno and colleagues reported two cases of indirect CCF (Barrow type B), a version of dAVF in which all arterial feeders were originated from ICA, that were successfully treated with the Pipeline Flex embolisation device with shield technology (Medtronic).^14^ Endothelialisation of the flow-diverting stent allowed for occlusion of the many arterial branches feeding the fistula.

## Transvenous approach

Before the Onyx era, transvenous approach was the mainstay of endovascular treatment for cure of dAVF given that transarterial embolisation was successful only in about 50% of cases.^22 32^ In a modern transvenous approach, the affected sinus and affected cortical vein are retrogradely catheterised and are occluded using microcoils, liquid embolic agents or their combination. For transvenous approach, appropriate patient selection is crucial to achieve complete occlusion and to avoid complications. The selected sinus or cortical vein should be wholly involved in drainage of the fistula and without participation in normal venous drainage of the brain, and this venous pathway should be completely occluded for proper treatment.^2^

Transvenous approach is preferred when a dAVF is supplied by small tortuous arteries excluding safe transarterial access to fistulous part, when dAVF is only supplied by branches directly from the ICA or vertebral artery, when dAVF is supplied by arteries with dangerous extracranial to intracranial anastomosis, or when the dAVF is supplied by nutrient arteries of cranial nerves.^33 34^ Similarly, a transvenous approach is the first line of treatment of an indirect CCF, where there is high probability of dangerous anastomosis, involvement of arteries supplying cranial nerves and very small feeding arteries to the fistula.^34 35^ Type I and II dAVFs of the hypoglossal canal are also the very good candidates for transvenous embolisation.^36^

Various routes to achieve transvenous access are used. Transvenous access can be established through the femoral vein, internal jugular vein, or direct puncture to affected sinus by burr hole, craniotomy, or ultrasound-guided puncture of a pericranial venous pouch.^37 38^ In situations of a trapped or thrombosed sinus/vein, a transvenous approach can be very challenging. In these cases, access could be achieved by crossing or recanalisation of a closed venous segment. Examples include traversing an ipsilateral occluded/thrombosed inferior petrosal sinus to access a trapped fistulous pouch of the cavernous sinus or traversing an occluded/thrombosed sigmoid sinus for approaching an isolated transverse sinus.^39 40^ In this approach, the interventionalist needs to carefully manage the risk of catheter or wire perforation of the thrombosed sinus or vein.^40^ Alternatively, sometimes a contralateral approach is possible. For example, contralateral jugular-sigmoid system across the torcula can be used.^41^ Direct access of trapped sinus or fistula pouch could be accomplished by direct puncture through a burr hole or craniotomy.^37^ Direct access of trapped cavernous pouch in indirect CCF can be fulfilled via the foramen ovale puncture or transorbital puncture.^42 43^ Hybrid angio-operating rooms are ideal for this sort of combined endovascular-surgical approaches.

Coil embolisation with platinum microcoils is highly effective for packing and occlusion of an affected sinus or venous pouch, particularly an isolated transverse sinus.^19 33^ Onyx is sometimes required in combination with coil to help seal the involved sinus or venous pouch. Onyx can be injected via transarterial approach or transvenous approach depending on the fistula anatomy.^33 44^ Some authors prefer ‘double-catheter technique’, one proximal microcatheter for deploying coils and, second, distal microcatheter for injecting Onyx after coiling.^33^ To avoid progression of retrograde thrombosis to normal cortical veins, some practitioners recommend anticoagulation for a few days after sacrifice of a trapped transverse sinus.^39^

While transvenous Onyx embolisation is reported in small case series of dAVFs it is the mainstay of treatment for indirect CCF.^33 45^ For ethmoidal and anterior cranial fossa dAVF, feeding arteries are often small and very tortuous leading to difficult or impossible safe superselective transarterial catheterisation.^46^ In these cases, transvenous Onyx embolisation may be safe when there is a non-tortuous draining vein allowing catheter navigation into the venous pouch.^46 47^ Albuquerque and colleagues described transvenous Onyx embolisation for treatment of high-risk transverse-sigmoid sinus dAVF in which microcatheter was navigated transvenously into the venous pouch and positioned into arterial ostium.^46^ In these cases, Onyx penetrated into multiple arterial feeders with small reflux into the venous pouch.^46^ This technique is reported to be safe when there is venous pouch or isolated sinus.

When a dAVF directly drains into the transverse and/or sigmoid sinuses and there is no venous pouch, there is high chance of Onyx reflux to normal sinus. Significant reflux into a normal sinus can lead to pulmonary embolism or inadvertent sinus thrombosis. In these situations, the reconstructive transvenous balloon-assisted embolisation (Onyx tunnel technique) was introduced as an option. For this technique, a microcatheter and a compliant balloon are simultaneously navigated into the transverse sinus and placed in the distal end of dAVF.^48^ After inflation of the balloon, Onyx 18 was injected at the periphery of the balloon to penetrate slowly into feeding arteries resulting in an Onyx tunnel leading to complete occlusion of dAVFs.^48^ In this technique, preservation of normal cortical veins, such as vein of Labbé, is essential to prevent complications such as venous infarction and cerebral haemorrhage. A remodelling balloon can also be used in the main draining vein to temporarily block the antegrade venous drainage during Onyx embolisation of fistulous venous pouch.^49^ When venous drainage of the brain is dependent on a fistulous transverse sinus, reconstruction of the transverse sinus using a stent with or without transarterial Onyx embolisation could be a good option.^50^

Major complications of a transvenous approach include vessel perforation (particularly during navigation of the wire or catheter), cerebral haemorrhage and venous infarction.^33 40 46 51^ Cranial nerve injury can occur during navigation or secondary to thrombosis or overpacking and involved venous region.^52^ Visual loss, glaucoma and acute exophthalmos were reported following transvenous approach via superior orbital vein for treatment of indirect CCF.^53 54^ Following transvenous embolisation of dAVF, venous drainage of normal brain could alter and lead to intracranial hypertension that can present with worsening of headache, confusion and neurological deficit.^51^ Mediastinal perforation, cardiac embolism and pulmonary embolism are the other potential complications of transvenous approach.^46 51^ If the fistula has pial arterial supply, in addition to dural arterial supply, sole reliance on a transvenous approach without retrograde penetration of pial feeders can result in haemorrhage. Similarly, if a fistula has CVR, a transvenous approach without retrograde penetration of feeding arteries can potentially convert the fistula to a higher grade with worsening cortical vein involvement.

## Stereotactic radiosurgery

Stereotactic radiosurgery (SRS) is usually reserved as a last salvage option for treatment of dAVF. Endothelial cell damage and thrombosis are suggested as the main mechanisms of dAVF occlusion by radiation.^55^ As with SRS for treatment of cerebral AVMs, obliteration of a dAVF could take many months, and during this latent period the risk of haemorrhage remains.^56–59^ Accordingly, low-risk dAVFs (Cognard grade 1 or Borden grade 1) could be good candidates for SRS. They showed a higher rate of obliteration by radiosurgery without haemorrhage during the latent period.^56^ It can also be a complementary treatment when endovascular therapy and surgery have failed.^56^ SRS can be used for high-grade AVF when endovascular or surgical approaches are too dangerous or have failed.^55^ Complete obliteration is reported in 50%–93% of dAVF, treated by SRS.^56–58 60^ SRS results in higher rates of complete obliteration and symptom improvement in indirect CCFs than in dAVFs of the transverse and sigmoid sinuses.^57^ The average latency period of dAVF closure following SRS was reported as 23 months.^59^ After SRS for dAVF, annual rate of rebleeding was reported as high as 2.6%,^60^ but this will depend on the starting fistula grade. For follow-up, annual MRI is recommended and angiography is mandatory for accurate diagnosis of complete dAVF obliteration.^56 57^ Complications include cranial nerve palsy, brain oedema, haemorrhage during the latency period and radiation effect.^56 57 60^

## Surgery

While endovascular techniques are often considered first-line therapy for treatment of dAVFs, surgery remains an alternate effective and safe option.^61^ For dAVF of the transverse-sigmoid sinus, surgery involves a wide exposure and skeletonisation of the involved sinus by isolation and coagulation of dural arterial feeders and arterialised cortical veins.^61^ The affected sinus can be completely removed if it is non-functional and does not participate in venous drainage of the normal brain. Care must be taken during surgery to avoid significant blood loss, particularly in complex fistulas with highly developed and hypertrophied networks of arterial feeders. In non-sinus dAVF, the cortical draining vein is disconnected from the fistula point by clips or coagulation.^61 62^ Frameless stereotactic navigation is helpful to localise the fistulous connection during craniotomy.^63^ Occlusion of dAVF during surgery is usually confirmed by indocyanine green angiography with or without intraoperative DSA.^64^

In few locations, such as anterior cranial fossa and ethmoidal dAVF, surgery is thought to be more successful than endovascular approaches.^65^ However, surgery is usually reserved for cases in which endovascular approaches have failed to completely cure the lesion.^66^ Long-term morbidity and mortality of dAVF surgery are reported to be as high as 13%.^63^ The major complications of surgery include infection, hydrocephalus, cerebrospinal fluid leak, stroke, cranial nerve palsy and severe blood loss.^63^ Preoperative embolisation can be helpful to reduce surgical blood loss.

## Conclusions

Endovascular treatment is generally the first-line treatment for dAVF. Prior to intervention, a complete understanding of the fistula angioarchitecture is required. This involves identification of feeding arteries, the fistula connection point, venous drainage pathways and the direction of venous flow. Endovascular approaches are performed from arterial, venous or combined pathways depending on the location and anatomy of a fistula. Surgery and to a lesser extent SRS remain as alternative treatment options, particularly when an endovascular approach is unsuccessful or considered dangerous. Follow-up angiography is required to confirm long-term occlusion of dAVF and the durability of treatment.
